# Sero-prevalence of brucellosis among slaughterhouse workers in Bahr el Ghazal region, South Sudan

**DOI:** 10.1186/s12879-019-4066-4

**Published:** 2019-05-22

**Authors:** Nuol Aywel Madut, Moses Ocan, Adrian Muwonge, John Bwalya Muma, George William Nasinyama, Jacques Godfroid, Ambrose Samuel Jubara, Clovice Kankya

**Affiliations:** 1 Department of Veterinary Clinical studies, Faculty of Veterinary Science Bahr el Ghazal University, South Sudan, P. O. Box 30, Wau, South Sudan; 2 Department of Biosecurity, Ecosystems &Veterinary Public Health (BEP). College of Vet. Animal Resources & Biosecurity (COVAB), Makerere University, P.O Box 7062, Kampala, Uganda; 3 Department of Pharmacology & Therapeutics, College of Health Sciences, Makerere University, P. O. Box, 7062 Kampala, Uganda; 4 Department of Genetics and Genomics, The Roslin Institute, University of Edinburgh, Edinburgh, Scotland; 5 Department of Disease Control, School of Veterinary Medicine, University of Zambia, P.O. Box 32379, Lusaka, Zambia; 6 Department of food Safety and infection Biology, Norwegian School of Veterinary Science, 9010 Troms∅, Norway

**Keywords:** Brucellosis, Slaughterhouse worker, Sero-prevalence, South Sudan

## Abstract

**Background:**

Brucellosis is an infectious zoonotic disease and is common especially among pastoral communities in most low and middle-income countries. The aim of this study was to determine sero-prevalence, and risk factors of Brucella infection among Slaughterhouse workers, in Bahr el Ghazal region, South Sudan.

**Methods:**

A cross sectional study was conducted among Slaughterhouse workers in Bahr el Ghazal region, South Sudan from December 2015 to May 2016. A pre-tested questionnaire was used in data collection. Each randomly selected participant was interviewed and a venous blood sample collected. The blood samples were screened for Brucellosis infection using Rose Bengal Plate Test (RBPT) and confirmed using Competitive Enzyme link Immuno Sorbet Assay (c-ELISA). Data was analyzed using Stata version 13 at 95% level of confidence.

**Results:**

A total of 234 Slaughterhouse workers were screen for Brucella infection. Overall, a third, 32.1% (75/234) of the participants were sero-positive for brucellosis. The prevalence of brucellosis was higher, 17.1% (40/234) in Wau state compared to other states. There was high prevalence among males, 28.6% (67/234) compared to females 3.4% (8/234). The mean age of study participants was 34.4 ± 9.6 years. A high proportion, 12.8% (30/234) of participants with confirmed brucellosis infection were 31–40 years of age. Brucellosis prevalence was high among butchers, 14.5% (34/234), and meat handlers, 9.0% (21/234).

**Conclusions:**

Brucellosis is common among animal slaughterhouse workers in Bahr el Ghazal region, South Sudan. There is need for public awareness campaigns and educational programs to help sensitize communities on Brucella infection.

## Background

Brucellosis is an infectious zoonotic disease caused by *Brucella* [1]. Humans get infected with Brucella through consumption of infected animal products, direct contact with infected animal secretions and excreta [2]. In some cases, brucellosis is acquired directly through contact with contaminated laboratory materials (specimens or cultures) during diagnostic or vaccination procedures [3].

Brucellosis is prevalent globally especially among pastoral and agro pastoral communities. A study by McDermott & Arimi, (2002) reported 5% prevalence of Brucellosis in Sub-Saharan Africa. The disease is mostly misdiagnosed and/or underreported among humans in most low and middle-income countries. This is likely to contribute to spread of the disease due to limited awareness and inadequate health care infrastructure especially in low and middle income countries [4]. Brucellosis has been reported among nomads, veterinary staff, abattoir workers and butchers [5]. A previous study by Hashim, 2007 reported positive Brucella test among abattoir workers in Omdurman city slaughterhouse in Sudan [6].

Brucella grows intracellularly producing variable bacteremia phases followed by localization of infections in tissues of the genital tract, mammary glands and reticulo-endothelial system [7]. *Brucella* species known to cause human Brucellosis include *Brucella melitensis*, *Brucella suis, Brucella abortus and Brucella canis*. Brucellosis is diagnosed by isolation of Brucella organism or by a combination of serological tests and clinical findings [8]. Human brucellosis cannot be diagnosed solely based on clinical grounds due to a wide variety of clinical manifestations. In addition, performing bacteriological culture take long time and the bacteria some time difficult to recover from the samples. This leaves use of serological tests as the only viable means of screening and confirming Brucellosis in clinical samples. The Rose Bengal plate test can be used as a sensitive rapid screening test but results should be confirmed by bacteriological and other serological tests. Tests like serum (tube) agglutination test (SAT), or micro-titre plate and Enzyme-linked immunosorbent assay (ELISA), for directions of antibodies like IgM and IgG [3].

Human brucellosis can be prevented through control of animal brucellosis using vaccines, and imposing fastidious hygiene measures on handling and processing of animal products. Effective use of preventive strategies like vaccination programs and education of communities has helped significantly in reducing the prevalence of Brucellosis especially in high-income countries [9]. However, the main challenge of human Brucella treatment is inadequate laboratory diagnosis. This is further complicated by Brucellosis having symptoms similar to malaria, a common disease in Sub-Saharan Africa. As such, there is high likelihood of misdiagnosis and potentially inappropriate treatment. Which could delay patients from obtaining definitive treatment, and thus worsen the disease and increase risk of unwanted outcomes such as death.

Individuals working in slaughterhouses are likely to have an increased risk of getting Brucella infection especially in endemic areas. However, in South Sudan the prevalence of Brucellosis is not known especially among high-risk groups such as veterinary staff, pastoralists and animal slaughterhouse employees. This study was thus intended to establish the prevalence of Brucellosis and associated risk factors among slaughterhouse workers in Greater Bahr el Ghazal, South Sudan.

## Methods

### Study area

The study was done in Greater Bahr el Ghazal region which is located northwest of the capital Juba. Four states (Gogrial, Tonj, North and Western Bahr el Ghazal) were randomly selected. From each state, one city was purposively selected (Tonj, Kuajok, Aweil and wau) where an abattoir per city was randomly selected.

### Study population

The study was conducted among individuals who work in abattoirs in Bahr el Ghazal region, South Sudan. Each of the abattoirs has about 30-to-80 employees. Veterinary staff or community health workers supervise each slaughterhouse.

### Study design and sample size

This was a cross sectional study. A sample size of 138 individual was estimated using a standard formula for cross-sectional studies [10]. In calculating the sample size, prevalence of Brucellosis in the study area was assumed to be 10% as reported from a previous study [11], 95% level of significance and a Z value of 1.96.

### Data collection methods

Data was collected using interviewer administered questionnaire and laboratory experiments. Variables in the questionnaire included; socio-demographic information (gender, marital status, religious affiliation, education attainment, occupation and ethnicity), location/area where participant comes from, and annual general medical check-up. A structured questionnaire was developed using information from literature and was pre-tested on 20 individuals from Juba town. Information from the pre-test was used in adjusting the tool. Five research assistants who were fluent in the local language and English were trained for field data collection using the study questionnaire. Laboratory data was collected using serological tests RBPT and c-ELISA. This method according to [12, 13].

### Sampling criteria and sample collection

In each of the selected abattoirs, employees were recruited using consecutive sampling method. 5 ml of venous blood was collected into purple top vacutainer bottles, which were kept at room temperature tilted at an angle of 45°c overnight to allow for clotting. The sera from vacutainer tubes were pipetted and transferred to a new set of Eppendorf tubes. The tubes were then uniquely labeled, stored on ice pack and transferred to EPI center in Wau teaching hospital laboratory where they were kept at -80 °C. After collecting all the samples, the serum was then transported to the central laboratory at Makerere University, College of Veterinary Medicine and Biosecurity. At the central laboratory, the serum samples were stored at -80 °C until analysis.

### Laboratory methods

#### Rose Bengal plate test

This was done following a procedure reported in a previous study [14]. Briefly, samples (serum and antigen) were brought to room temperature. 30 Microliter of each serum sample was placed on a white plastic plate. After shaking the antigen bottle, an equal volume of the antigen was added on the plastic plate. These were then mixed uniformly using a clean glass rod. The mixture was agitated gently for 4 min at an ambient temperature on a rocker, after which the agglutination was read. Any visible agglutination was considered positive.

### Enzyme linked immune sorbent assay (ELISA) competitive ELISA

The Brucellosis Antibody Test Kit (IDEXX – laboratories, Italia) and positive control Brucellosis Serum (P04130–13) used in this study were adopted from a study by [15, 16]. The test was performed following manufacturer’s guidelines. The results were reported as negative, suspected, or positive when the ratio of sample optical density to the positive –control optical density (S/P ratio) was less than 70, 70to 100%, more than 100%, respectively.

### Data analysis

Data analysis was done, using Stata version 13. Chi-square test was used and statistical significance was taken as *P* < 0.05.

## Results

### Socio-demographic characteristics of animal slaughterhouse workers

A total of 234 Slaughterhouse workers were screened for Brucella antibodies. The majority, 89.3% (209/234) of animal slaughterhouse workers were males. The mean age of study participants was 34.4 ± 9.6 years. The majority of respondents, 73.9% (173/234) were married. Most, 43.6% (102/234) of the slaughterhouse workers didn’t have any formal education. The majority, 40.2% (94/234) of animal slaughterhouse workers were butchers. Over half, 50.6% (119/234) of the animal slaughterhouse workers were Nilotic (Table 1).

**Table 1:** Demographic characteristics of animal slaughterhouse workers

Factor	Description	Total N (%)	RBPT N (%)	C- Elisa N (%)	*P*-Value
Sex	Female	25 (10.7)	9 (36)	8 (32)	**0.995**
	Male	209 (89.3)	89 (42.6)	67 (32.1)	
Age	10–20	25 (10.7)	12 (48)	5 (20)	**0.242**
	21–30	80 (34.2)	33 (41.3)	23 (28.8)	
	31–40	89 (38.0)	33 (37.1)	30 (33.7)	
	> 40	40 (17.1)	20 (50)	17 (42.5)	
Marital Status	Married	173 (73.93)	70 (40.5)	62 (35.8)	**0.101**
	Single	61 (26.1)	28 (19.7)	13 (21.3)	
Education	No formal education	102 (43.6)	39 (38.2)	33 (32.3)	**0.420**
	Tertiary education	12 (5.1)	4 (33.3)	3 (25)	
	Primary education	98 (49.9)	46 (46.9)	33 (33.8)	
	Secondary education	22 (9.4)	9 (40.9)	6 (27.3)	
Religion	Christian	150 (64.1)	66 (44)	52 (34.7)	**0.252**
	Muslim	84 (35.9)	32 (38.1)	23 (27.4)	
Occupation	Vet assistant	21 (9.0)	12 (57.1)	10 (47.6)	0. 004*
	Butcher	94 (40.2)	45 (47.9)	34 (36.2)	
	Health worker	14 (6.0)	9 (64.3)	5 (35.7)	
	Meat handler	57 (24.4)	25 (43.9)	21 (36.8)	
	Administrator	4 (1.7)	2 (50)	2 (50)	
	*Others	44 (18.8)	5 (11.4)	3 (6.8)	
Ethnicity	Nilotic	119 (50.9)	48 (40.3)	34 (28.6)	0.033*
	Luo	5 (2.1)	3 (60)	3 (60)	
	Bantu	42 (17.9)	24 (57.1)	21 (50)	
	Jur	43 (18.4)	16 (37.2)	13 (30.2)	
	Arab	24 (10.3)	6 (25)	3 (12.5)	
	Nubba	1 (0.4)	1 (0.4)	1 (0.4)	
Locality	Aweil	57 (24.4)	12 (21.1)	12 (21.1)	0.027*
	Kuajok	32 (13.7)	32 (13.7)	13 (40.6)	
	Tonj	45 (19.2)	10 (22.2)	10 (22.2)	
	Wau	100 (42.7)	44 (44)	40 (40)	
Total		**234**	**98 (41.9)**	**75 (32.1)**	

*Others include: Casual workers, cooks and animals traders

### Sero-prevalence of brucellosis among animal slaughterhouse workers in Bahr el Ghazal region, South Sudan

A third, 32.1% (75/234) of animal slaughterhouse workers in Bahr el Ghazal region had Brucella Antibodies. Sero-prevalence of Brucellosis was high among slaughterhouse workers from Wau state, 18.8% (44/234). Slaughterhouse workers who were 31-to-40 years of age had a higher prevalence of Brucella antibodies, 12.8% (30/234). Most, 22.2% (52/234) of the butchers had Brucella infection. The rate of Brucella antibodies was higher, 42.6% among males compared to females 36%. Brucella antibodies was higher among the married animal slaughterhouse workers, 26.5% (62/234) (Table 1).

### Factors associated with Brucella infection among animal slaughterhouse workers in Bahr el Ghazal region, South Sudan

The factors significantly associated with Brucella sero-prevalence included; occupation (*P* = 0.04), Ethnicity (*P* = 0.033), locality (*P* = 0.027) (Table 1). Annual medical checkup (*P* = 0.049) had boarder line association with Brucella infection (Table 2).

**Table 2:** Brucella sero – prevalence versus risk factors and known symptoms

Risk factors	Response	Frequency *N* = 234 (%)	RBPT *N* = 98 (%)	C-ELISA *N* = 75 (%)	*P*-value
Has hand abrasions	Yes	60 (25.6)	59 (37.6)	52 (33.1)	0.709
	No	172 (73.5)	39 (52.0)	23 (30.7)	
Wash hands after work	Yes	157 (67.1)	81 (46.0)	59 (33.5)	0.229
	No	75 (32.1)	13 (26.5)	12 (24.5)	
*Consumption of animal products	Yes	198 (84.62)	14 (38.89)	63 (31.82)	0.858
	No	36 (15.38)	84 (42.42)	12 (33.33)	
Knowledge of zoonotic diseases	Yes	83 (35.5)	40 (48.2)	43 (28.5)	0.114
	No	151 (64.5)	58 (38.4)	32 (38.6)	
Health complain					
Fever	Yes	69 (29.5)	31 (44.9)	23 (33.3)	0.786
	No	165 (70.5)	67 (40.6)	52 (31.5)	
Headache	Yes	62 (26.5)	27 (43.5)	22 (35.5)	0.499
	No	172 (73.5)	71 (41.3)	53 (30.8)	
Joint Pain	Yes	42 (17.95)	19 (45.2)	15 (35.7)	0.574
	No	192 (82.05)	79 (41.1)	60 (31.2)	
Fatigue	Yes	54 (23.1)	23 (42.6)	18 (33.3)	0.818
	No	180 (76.9)	75 (41.7)	57 (31.7)	
Night sweating	Yes	31 (13.25)	10 (32.3)	9 (29)	0.699
	No	203 (86.75)	88 (43.35)	66 (32.5)	
Annual Medical Check up	No	172 (73.5)	67 (38.9)	49 (28.5)	0.049*
	Yes	59 (25.2)	29 (49.1)	25 (42.4)	
Use Personal Protective gear	No	158 (68.1)	66 (41.8)	46 (29.1)	0.126
Duration at work	Yes	74 (31.9)	32 (43.2)	29 (39.2)	
	< 5	128 (55.4)	57 (58.8)	39 (52.7)	0. 570
	≥ 5	103 (44.6)	40 (41.2)	35 (47.3)	

* Meat, milk and urine

On bivariate analysis, Nilotic slaughterhouse workers were twice more likely to have Brucella infection compared to Arab slaughterhouse workers (Table 3).

**Table 3:** Bivariate analysis of factors associated with brucellosis

Factor/variable	Description	Adjusted OR	*P*-value	95% C.I.
Annual Medical Checkup	Yes	1	–	–
	No	0. 54	0.050*	0 .29–1.00
Locality	Wau	1	–	–
	Aweil	0 .4	0.017*	0 .19–0.85
	Kuajok	1.03	0.950	0.46–2.31
	Tonj	0 .43	0.040*	0 .19–0.96
Occupation	Administrator	1	–	–
	Vet/ assistant	0.91	0.930	0.11, 7.72
	Butcher	0.57	0.579	0.076, 4.21
	Health worker	0.56	0.608	0.06, 5.24
	Meat handler	0.58	0.603	0.08, 4.45
	*Others	0.07	0.025*	0.01, 0.71
Ethnic Group	Arab	1	–	–
	Nilotic	2.53	0.155	0.70, 9.12
	Luo	9.5	0.041*	1.09, 82.72
	Bantu	6.33	0.008*	1.63, 24.67
	Jur	2.74	0.152	0.69, 10.92

*Others include: Casual workers, Cooks (Tea/food) and cattle traders;

*OR* Odds Ratio

On Multivariate analysis of the risk factors of Brucella infection, there were no significant predictors of Brucella sero-prevalence among animal slaughterhouse workers in Bahr el Ghazal region (Table 4).

**Table 4:** Multivariate logistic regression analysis of the predictors of Brucellosis

Predictors	Description	OR	*P*-value	95% C.I.
Annual Medical Checkup	Yes	1		
	No	0.80	0.589	0.35, 1.82
Locality	Wau	1		
	Aweil	0.59	0.456	0.15, 2.36
	Kuajok	0.97	0.956	0.28,3.28
	Tonj	0.47	0.264	0.12, 1.77
Occupation	Administrator	1		
	Vet/assistant	1.26	0.842	0.13, 12.11
	Butcher	1.14	0.904	0.13, 9.81
	Health worker	0.76	0.820	0.08, 7.76
	Meat handler	0.87	0.900	0.10, 7.31
	Others	0.167	0.154	0.015, 1.95
Ethnic Group	Arab	1		
	Nilotic	5.26	0.033	1.14, 24.33
	Luo	8.66	0.068	0.85, 88.27
	Bantu	5.56	0.021	1.30, 23.83
	Jur	2.70	0.171	0.65, 11.19

## Discussion

In this study, total of 234 Slaughterhouse workers were screened for Brucella antibodies, from slaughterhouses in Bahr el Ghazal region. There were more male slaughterhouse workers than females. This could be due to the working conditions in the slaughterhouse especially the amount of effort or labor required. In this study the average age of workers was 34.5 years, a finding similar to that of previous studies [17, 18].

The study found a third of animal slaughterhouse workers in Bahr el Ghazal to be having Brucellosis. This is similar to 35.7% prevalence reported in previous studies [17, 19]. The prevalence rate in our study was higher than that the 8% reported in India and Sudan [20–22]. The poor condition of animal slaughterhouses in South Sudan and lack of regulatory framework could have contributed to the high risk of contracting Brucella infection among animal slaughterhouse workers. This finding is important especially for health care providers as it helps draw attention to Brucellosis as a common disease in the region. Other endemic diseases like malaria in the region may complicate Brucella diagnosis in the region. There is a high risk of mistreatment as a result of inadequate diagnosis. This is especially likely as most physicians manage patients based on clinical presentation. As a result, most patients may delay in getting appropriate treatment and thus worsening of disease condition, increasing cost of care and potentially lead to death.

Brucella sero-prevalence was high in Wau state compared to other states in Bahr-el-Ghazal region, South Sudan. This could be attributed to the large number of animals slaughtered in Wau since it previously acted as the capital before creation of new states and is still one of the largest cities in South Sudan. Brucellosis sero-prevalence was high among screened males compared to females. This could be due to high exposure to contaminated animal products among males due to their involvement in most slaughterhouse activities compared to females [23]. Furthermore inadequate hygiene practices and poor adherence to correct use of protective gears could potentially increase the risk of infection and may explain the high prevalence of Brucellosis among slaughterhouse workers reported in this study [17].

In this study, Brucellosis prevalence was higher among butchers, meat handlers and veterinarians. This finding is similar to that of previous studies [17, 24] and could be attributed to the increased contact with infected animals products in the slaughterhouse. Consumption of raw animal products like meat, milk and urine was associated with Brucellosis infections [24].

### Limitation of the study

As a result of the Instability in the region due to the civil war, we had to restrict the study to only four cities, which were stable at the time of data collection.

## Conclusions

A third of the animal slaughterhouse workers in Bahr el Ghazal have Brucellosis infection. There is need to sensitize animal Slaughterhouse workers about zoonotic diseases, and enforcement of hygiene practices and use of protective gear. In addition, the health workers in hospitals need to be sensitized to increase screening for Brucellosis among patients presenting to hospitals especially with symptoms similar to those of malaria like fever, a common disease in the region.

## Abbreviations

C-ELISA: Competitive enzyme-linked immuno sorbent assay
CI: Confidence interval
RBPT: Rose bengal plate agglutination test
